# Pulmonary Vascular Remodeling and Prognosis in Patients Evaluated for Heart Transplantation: Insights from the OCTOPUS-CHF Study

**DOI:** 10.3390/jcdd9120439

**Published:** 2022-12-06

**Authors:** Jorge Martínez-Solano, Enrique Gutiérrez-Ibañes, Carlos Ortiz-Bautista, María Dolores García-Cosío, Fernando Sarnago-Cebada, Beatriz Díaz-Molina, Isaac Pascual, Juan Francisco Oteo-Domínguez, Manuel Gómez-Bueno, Ramón Calviño-Santos, María G. Crespo-Leiro, Joan Antoni Gómez-Hospital, Carles Díez-López, Juan García-Lara, Iris P. Garrido-Bravo, Luis de la Fuente-Galán, Javier López-Díaz, Sonia Mirabet-Pérez, Manuel Martínez-Sellés

**Affiliations:** 1 Servicio de Cardiología, Hospital General Universitario Gregorio Marañón, Instituto de Investigación Sanitaria Gregorio Marañón (IISGM), 28007 Madrid, Spain; 2 Centro de Investigación en Red de Enfermedades Cardiovasculares (CIBERCV), Instituto de Salud Carlos III, 28026 Madrid, Spain; 3 Servicio de Cardiología, Hospital Universitario 12 de Octubre, Instituto de Investigación Sanitaria Hospital 12 de Octubre (IMAS12), 28041 Madrid, Spain; 4 Servicio de Cardiología, Hospital Universitario Central de Asturias, 33011 Oviedo, Spain; 5 Servicio de Cardiología, Hospital Universitario Puerta de Hierro Majadahonda, 28222 Madrid, Spain; 6 Servizo de Cardioloxía, Complexo Hospitalario Universitario A Coruña, 15006 A Coruña, Spain; 7 Servei de Cardiologia, Hospital Universitari de Bellvitge, 08907 Barcelona, Spain; 8 Servicio de Cardiología, Hospital Clínico Universitario Virgen de la Arrixaca, 30120 Murcia, Spain; 9 Servicio de Cardiología, Hospital Clínico Universitario de Valladolid, 47003 Valladolid, Spain; 10 Servei de Cardiologia, Hospital Universitario de la Santa Creu i Sant Pau, 08025 Barcelona, Spain; 11 Facultad de Ciencias Biomédicas y de la Salud, Universidad Europea, 28670 Madrid, Spain

**Keywords:** pulmonary hypertension, pulmonary vascular remodeling, advanced heart failure, optical coherence tomography, intravascular imaging, right heart catheterization, heart transplant

## Abstract

Objective: In patients with advanced heart failure, the intravascular optical coherence tomography (OCT) of subsegmental pulmonary artery measurements is correlated with right heart catheterization parameters. Our aim was to study the prognostic value of pulmonary OCT, right heart catheterization data, and the echocardiographic estimation of pulmonary pressure in patients studied for elective heart transplants. Methods: This research is an observational, prospective, multicenter study involving 90 adults with a one-year follow-up. Results: A total of 10 patients (11.1%) died due to worsening heart failure before heart transplantation, 50 underwent a heart transplant (55.6%), and 9 died in the first year after the transplant. The patients with and without events (mortality or heart failure-induced hospitalization) had similar data regarding echocardiography, right heart catheterization, and pulmonary OCT (with a median estimated pulmonary artery systolic pressure of 42.0 mmHg, interquartile range (IQR) of 30.3–50.0 vs. 47.0 mmHg, IQR 34.6–59.5 and *p* = 0.79, median pulmonary vascular resistance of 2.2 Wood units, IQR 1.3–3.7 vs. 2.0 Wood units, IQR 1.4–3.2 and *p* = 0.99, and a median pulmonary artery wall thickness of 0.2 ± 0.5 mm vs. 0.2 ± 0.6 mm and *p* = 0.87). Conclusion: Pulmonary vascular remodeling (evaluated with echocardiography, right heart catheterization, and pulmonary OCT) was not associated with prognosis in a selected sample of adults evaluated for elective heart transplants. Pulmonary OCT is safe and feasible for the evaluation of these patients.

## 1. Introduction

Pulmonary hypertension secondary to left-heart disease (PH-LHD) is highly prevalent and associated with an increased disease burden and poor outcomes [1,2,3]. The subset of patients with advanced heart failure (HF) comprises an estimated 5–10% of the overall HF population [4,5]. For these patients, a heart transplant (HT) is the treatment of choicein the absence of severe PH-LHD unresponsive to pharmacological intervention or other contraindications. A thorough characterization of pulmonary hypertension (PH) is crucial in the evaluation for HT candidacy [6]. Pulmonary vascular-remodeling estimation through right heart catheterization is still the gold standard but it entails interpretation variability, several technical drawbacks, and high load-dependency [7]. Furthermore, different studies in patients undergoing HT questioned its true association with the outcomes in this setting [8,9].

Optical coherence tomography (OCT) was first used in the field of retinal pathology but is now widely used in the diagnosis and treatment of coronary artery disease. OCT is based on a near-infrared light source with a resolution of 10–20 mm, which is particularly useful for tissue characterization [10,11]. Recently, OCT has emerged as a diagnostic tool in different subtypes of PH [12]. Pulmonary artery OCT showed no clear distinction between intima and media layers in previous studies, which described the vessel wall as a homogeneous, single-layered, and signal-rich structure [12]. The thickening of the wall by a structure with high reflectivity and low attenuation corresponds to fibrosis in pathological studies [13]. Previous studies in PH-LHD found a correlation between hemodynamics and OCT measurements of wall thickness (WT) [14,15], but data are lacking regarding its prognostic value in this subset of patients.

Our aim was to assess the prognostic value of pulmonary vascular remodeling in HF patients evaluated for an elective HT.

## 2. Materials and Methods

### 2.1. Study Design

Our data come from the Optical Coherence Tomography Observation of Pulmonary Ultra-Structural Changes in Heart Failure (OCTOPUS-CHF) study. The design of this study has already been published [16]. Briefly, 90 adults with advanced HF evaluated for HT were included, and pulmonary vascular remodeling was assessed with transthoracic echocardiography, right heart catheterization, and OCT of a subsegmental pulmonary artery. OCT was performed in the right-lower or the right-middle lobe (with a luminal diameter < 5 mm and minimal length of 50 mm). We excluded patients under mechanical circulatory support, with decompensated HF, shock, or those awaiting urgent HT. Significant comorbidities, such as chronic kidney disease, cancer, chronic obstructive pulmonary disease, or the presence of PH forms other than PH-LHD, were ruled out before inclusion. WT of 0.25 mm was set as the cutoff value for pulmonary vascular remodeling [15]. The primary endpoint included mortality and HF admission before and after HT.

### 2.2. Statistical Analysis

The normality of the continuous variables was studied via the Shapiro–Wilk test. Normally distributed variables are presented as mean ± standard deviation, and non-normal variables as median (interquartile range). Categorical variables are shown as absolute values and percentages. Student’s t test was used to compare normally distributed variables, Kruskal–Wallis test was employed for non-normal ones, and Chi-square test was used for categorical variables. All analyses were performed with R version 4.0.2 (R Core Team, Vienna, Austria) and SPSS 25.0 software (SPSS Inc., Chicago, IL, USA).

### 2.3. Ethical Aspects

The study followed the Declaration of Helsinki. The protocol was approved by the Ethics Committee of each participating center. All patients provided written informed consent.

## 3. Results

There were no serious immediate or long-term complications secondary to the procedure. Echocardiographic evaluations were carried out in 78 patients.

The one-year follow-up outcomes are depicted in Table 1. The median age was 57.5 years with men accounting for 78.9% of the sample. A total of 19 patients died (21.1%), of which 10 patients (11.1%) died due to worsening HF before the HT. Fifty patients underwent a HT (55.6%), and nine died within the first year (six from severe primary graft failure, one from acute graft rejection, one from complicated respiratory failure secondary to coronavirus infection, and one from a hemorrhagic stroke).

**Figure 1:**
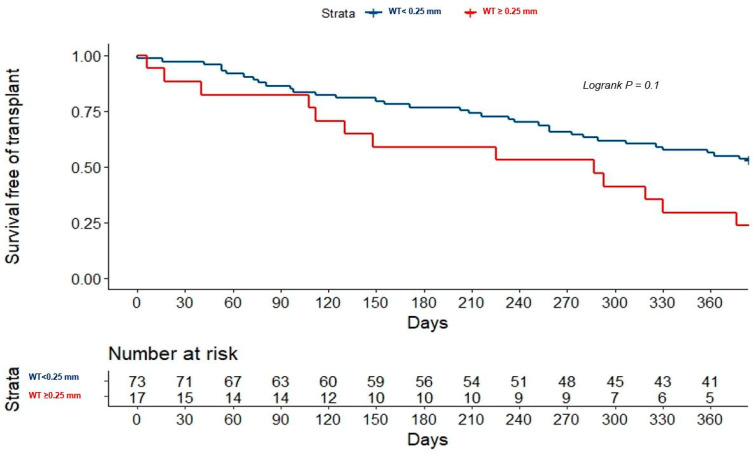
Kaplan–Meier curves for death or heart transplantation according to pulmonary OCT results. OCT: optical coherence tomography, WT: wall thickness.

**Figure 2:**
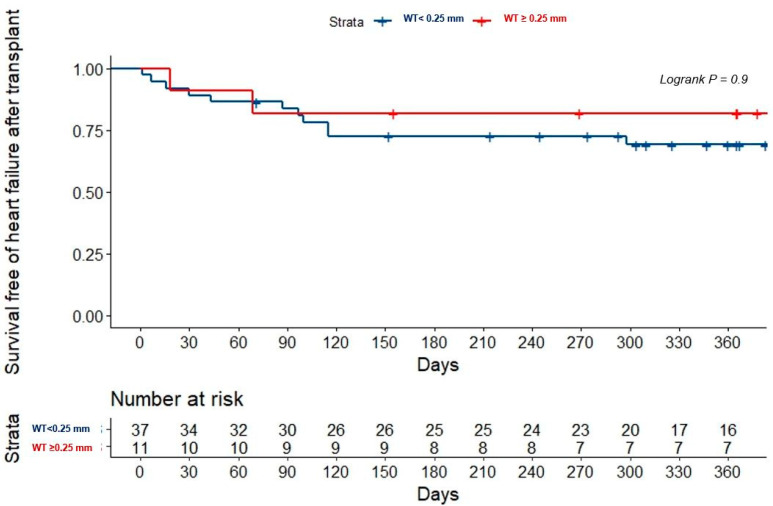
Kaplan–Meier curves for death or heart failure admission after heart transplantation regarding pulmonary OCT results. OCT: optical coherence tomography, WT: wall thickness.

The PH data from echocardiography, right heart catheterization, and OCT were similar between the patients that met the primary endpoint (death or HF admission) and those who did not (median estimated pulmonary artery systolic pressure of 42.0 mmHg, interquartile range (IQR) of 30.3–50.0 vs. 47.0 mmHg, IQR of 34.6–59.5 with *p* = 0.79, median pulmonary vascular resistance of 2.2 Wood units, IQR of 1.3–3.7 vs. 2.0 Wood units, IQR of 1.4–3.2 with *p* = 0.99, and median pulmonary artery wall thickness 0.2 ± 0.5 mm vs. 0.2 ± 0.6 mm with *p* = 0.87). The primary endpoint was associated with male sex, an INTEragency Registry for Mechanically Assisted Circulatory Support (INTERMACS) status of 3–4, atrial fibrillation, and significant mitral regurgitation (Table 2), but only INTERMACS predicted the primary endpoint in the multivariate analysis (Table 3). Pulmonary vascular remodeling was not associated with outcomes in either the global population (Table 2 and Figure 1) or in the HT recipients (Table 4 and Figure 2). 

## 4. Discussion

In the present multicenter, observational study, we sought to evaluate the prognostic role of the in vivo assessment of pulmonary vascular remodeling with different techniques—including pulmonary OCT—in advanced HF patients referred for an elective HT. The use of pulmonary OCT was feasible and safe for these patients, with no severe complications during the procedure and after a one-year follow-up. Our main finding is that there were no significant differences in the pulmonary vascular remodeling measurements (evaluated with OCT, right heart catheterization, and echocardiography) between the patients who met the primary endpoint (HF admission and mortality) and those who did not. 

Across the spectrum of HF, more than 50% of the cases present with secondary PH; this value is virtually 100% in the case of end-stage disease [1,4]. Once established, PH plays an independent and central role, even when the underlying condition has been corrected [17,18], leading to more severe symptoms, poorer exercise tolerance, and higher mortality [2,3]. Despite the enormous prognostic relevance of PH-LHD, the biological and physiological bases remain poorly understood and come from the field of primary PH. Currently, specific pathways through which to target are lacking, so there is an unmet need for the deeper comprehensive profiling of PH-LHD [1].

Right heart catheterization is still the cornerstone for a PH assessment. However, a knowledge gap around its interpretation has been reported [1]. This procedure entails technical challenges and waveform analysis variability [7]. Moreover, it assumes indirect estimations of pulmonary vascular remodeling given its steady-flow view and load dependency [1,19,20,21]. Despite these limitations, hemodynamic data—such as pulmonary artery pressure, transpulmonary gradient, and, especially, pulmonary vascular resistance—have demonstrated their prognostic utility in non-selected HF patients [22,23,24,25]. Nonetheless, conflicting results have been published, since these measurements are highly dependent on pulmonary wedge pressure [26,27] and have failed to prove their suitability to guide HF therapy [28]. Interestingly, a post-mortem study in HT recipients found that pulmonary arteries were thicker than expected based on the pretransplant right heart catheterization results [29]. Furthermore, there is uncertainty regarding the appropriate cutoff values and their significance in the decision-making process regarding a specific patient with advanced HF [30]. Therefore, there is a growing need to find alternative methods for a more accurate evaluation of pulmonary vascular remodeling [19,21,30,31].

The morphological study of PH used to be achievable only through a pulmonary biopsy or necropsy; however, this changed with the recent rise of intravascular imaging techniques. Previous pulmonary OCT studies [12] reported associations with hemodynamic parameters in primary PH [32,33], chronic thromboembolic PH [34], and PH-LHD [14,15]. A study of primary PH found correlations between the WT area measured by OCT and hemodynamic parameters, pulmonary artery-related histology findings from the explanted lungs, and outcomes [13]. However, to the best of our knowledge, this is the first study to provide prognostic information on pulmonary OCT assessment among HF patients.

Retrospective studies on pulmonary hemodynamics of patients undergoing HT have generated concern about the risk of right ventricular failure and mortality among those patients with higher pulmonary vascular resistance and transpulmonary gradient [35,36,37,38,39]. In consequence, the suitability for an HT requires low pulmonary vascular resistance in compliance with those studies and international societies’ recommendations [6]. Recent studies found poor survival in non-selected adults with PH and a pulmonary resistance value over 2.2 Wood units [25]. Indeed, we found a statistically non-significant tendency to possess thicker wall vessels among those patients with higher mortality or requiring an HT (Figure 1). However, our results are not necessarily unexpected since most HT candidates display normal or slightly increased pulmonary vascular resistance. In fact, not only did the pulmonary OCT fail to prove its prognosis value, but so too did the echocardiographic and hemodynamic data. A recent meta-analysis found no association of PH with one-year mortality in a large sample of HT recipients [8]. In addition, a retrospective review of the United States organ transplantation database demonstrated a similar HT survival between patients with pretransplant pulmonary vascular resistance > 3 Wood units and transpulmonary gradient > 12 mmHg and in those with >5 Wood units and >15 mmHg [9]. Currently, it is widely accepted that pulmonary vascular resistance promptly decreases after the unloading of the left ventricle [40] or an HT [41]. The reverse remodeling of pulmonary vascular changes may explain why survival, at least beyond the first year of an HT, is not affected by pretransplant PH [41,42,43]. Our findings are in line with published evidence since pulmonary vascular remodeling assessed by OCT did not predict death or HF admission after an HT (Figure 2). 

However, this study has some limitations. Our small sample led to a low event rate and hampered the statistical power with which to show differences between subgroups. In addition, though pulmonary vascular remodeling is known to be more pronounced in the venous bed [44,45], the morphological evaluation of this side involves a higher risk of complications and has not been described yet. In addition, pulmonary OCT before and after an HT in the same patient was not performed but it could have shown the reverse remodeling process. Finally, all our patients had a clinical indication for right heart catheterization before an elective HT. This selection bias explains the absence of severe PH-LHD in our sample. Therefore, the clinical utility of pulmonary OCT cannot be ruled out after these results. Instead, pulmonary OCT’s safety and feasibility together with its proven correlation with hemodynamic data [12,15] may spark further investigations.

## 5. Conclusions

Pulmonary vascular remodeling evaluated with echocardiography, right heart catheterization, and pulmonary OCT were not associated with prognosis in this selected sample of adults evaluated for an HT. The clinical utility of OCT cannot be ruled out given the small size, absence of significant PH, and low event rate in this sample.

## Figures and Tables

**Table 1:** Baseline characteristics and events during follow-up.

	N = 90
Age (years)	57.5 [48.8–63.3]
Male sex	71 (78.9%)
HT	50 (55.6%)
HF readmission before HT	46 (51.1%)
Death before HT	10 (11.1%)
Primary graft failure	22 (24.4%)
ECMO implantation after HT	9 (10.0%)
Acute kidney injury after HT requiring RRT	9 (10.0%)
HF admission after HT	5 (5.6%)
Death after HT	9 (10.0%)

ECMO: extracorporeal membrane oxygenator. HF: heart failure. HT: heart transplant. RRT: renal replacement therapy.

**Table 2:** Clinical, echocardiographic, hemodynamic, and pulmonary OCT characteristics according to the development of events in the follow-up.

	Death or HF Admission	Alive without HF Admission	*p*
N	36	54	
Age	56.0 [44.3–61.0]	58.5 [50.5–65.3]	0.33
Male sex	24 (66.7%)	47 (87.0%)	0.02
Weight (kg)	77.0 [70.5–96.5]	79.0 [67.0–90.0]	0.40
Height (cm)	170.0 [160.0–179.0]	169.0 [166.0–175.0]	0.82
Underlying disease:			
Non-ischemic dilated cardiomyopathy	14 (38.9%)	19 (35.2%)	0.72
Ischemic heart disease	11 (30.6%)	19 (35.2%)	0.65
Valvular heart disease	0 (0.0%)	4 (7.4%)	0.09
Hypertrophic cardiomyopathy	7 (19.4%)	5 (9.3%)	0.16
Others	4 (11.1%)	7 (12.9%)	0.49
Heart failure drugs:			
Mineralocorticoid receptor antagonists	28 (77.8%)	41 (75.9%)	0.84
Beta-blockers	30 (83.3%)	37 (68.5%)	0.11
Angiotensin receptor blocker	5 (13.9%)	4 (7.4%)	0.32
Angiotensin-converting enzyme inhibitors	6 (16.7%)	6 (11.1%)	0.45
Sacubitril–Valsartan	19 (52.8%)	29 (53.7%)	0.93
NYHA class III–IV	28 (77.8%)	47 (87.0%)	0.25
INTERMACS status 3–4	10 (27.8%)	40 (74.1%)	0.001
Atrial fibrillation	13 (36.1%)	35 (64.8%)	0.007
Echocardiographic measurements:			
LVEF (%)	28.0 [20.0–35.0]	25.0 [18.0–32.0]	0.16
End-diastolic RV basal diameter	38.0 [33.0–45.5]	42.0 [37.0–47.0]	0.08
TAPSE (mm)	16.0 [12.0–18.8]	15.0 [13.0–18.0]	0.50
RV FAC (%)	35.0 [28.0–43.0]	33.0 [25.0–40.0]	0.51
PAAT (ms)	92.0 [62.8–119.5]	87.0 [70.0–98.5]	0.79
Significant mitral regurgitation	8 (22.2%)	25 (46.3%)	0.02
Significant tricuspid regurgitation	2 (5.6%)	7 (12.9%)	0.25
Non-invasively estimated sPAP (mmHg)	42.0 [30.3–50.0]	47.0 [34.6–59.5]	0.79
RHC measurements:			
mPAP (mmHg)	25.5 [17.8–38.1]	27.3 [19.6–31.2]	0.67
PWP (mmHg)	18.0 [12.0–24.0]	18.5 [13.0–25.0]	0.80
TPG	8.3 [5.1–12.4]	9.0 [5.7–11.7]	0.67
CO (L/min)	4.1 [3.6–4.6]	4.1 [3.4–4.6]	0.76
PVR (WU)	2.2 [1.3–3.7]	2.0 [1.4–3.2]	0.99
Pulmonary arterial compliance (mL/mmHg)	2.59 [1.70–3.94]	2.53 [1.65–3.49]	0.74
OCT measurements:			
Wall thickness (mm)	0.2 ± 0.5	0.2 ± 0.6	0.87
Wall thickness area (mm^2^)	2.4 ± 0.7	2.4 ± 0.9	0.81
Wall thickness index (%)	12.6 ± 2.8	12.6 ± 2.6	0.96
Wall thickness area index (%)	20.9 ± 3.8	20.9 ± 3.6	0.98

Values are shown as mean ± standard deviation for parametric continuous variables and median (interquartile range) for nonparametric ones. CO, cardiac output; INTERMACS, Interagency Registry for Mechanically Assisted Circulatory Support; FAC, fractional area change; HF, heart failure; LVEF, left ventricle ejection fraction; mPAP: medium pulmonary artery pressure; NYHA, New York Heart Association; OCT, optical coherence tomography; PAAT, pulmonary artery acceleration time; PWP, pulmonary wedge pressure; PVR, pulmonary vascular resistance; RV, right ventricle; TAPSE, tricuspid annular plane systolic excursion; TPG, transpulmonary gradient.

**Table 3:** Multivariate logistic regression analysis of primary endpoint predictors.

	OR (95% CI)	*p*
Age	1.00 (0.96–1.05)	0.83
Sex	2.91 (0.87–9.74)	0.08
INTERMACS status 3–4	5.54 (1.97–15.56)	0.01
Atrial fibrillation	2.49 (0.90–6.87)	0.08
Significant mitral regurgitation	2.09 (0.68–6.47)	0.20

CI, confidence interval; INTERMACS, Interagency Registry for Mechanically Assisted Circulatory Support; OR, Odds Ratio.

**Table 4:** Results of the patients that underwent heart transplantation to pulmonary artery wall thickness (WT).

	WT < 0.25 mm	WT ≥ 0.25 mm	*p*
N	38	11	
Age	56.0 [48.8–64.0]	55.0 [43.0–59.0]	0.76
Male sex	29 (76.3%)	9 (81.8%)	0.61
Weight (kg)	76.0 [66.0–87.0]	75.0 [64.0–100.0]	0.99
Height (cm)	168.0 [159.0–173.0]	171.0 [166.0–175.0]	0.10
NYHA class III–IV	34 (89.5%)	10 (90.9%)	0.98
INTERMACS status 3–4	29 (76.3%)	4 (36.4%)	0.01
Echocardiographic measurements:			
LVEF (%)	25.0 [19.8–34.3]	28.0 [17.0–53.0]	0.82
End-diastolic RV basal diameter	40.0 [31.5–45.3]	40.5 [35.3–45.3]	0.99
TAPSE (mm)	15.0 [12.0–17.8]	14.0 [12.8–17.3]	0.74
RV FAC (%)	33.0 [27.0–43.0]	29.0 [27.3–39.0]	0.78
PAAT (ms)	90.0 [71.5–97.5]	75.0 [64.3–117.5]	0.64
Severe mitral regurgitation	15 (39.5%)	4 (36.4%)	0.90
Severe tricuspid regurgitation	3 (7.9%)	1 (9.1%)	0.93
Non-invasively estimated sPAP (mmHg)	46.5 [31.0–57.0]	50.0 [37.0–65.0]	0.94
RHC measurements:			
RA (mmHg)	6.5 [4.0–10.3]	9.0 [5.5–14.0]	0.47
sPAP (mmHg)	37.0 [30.8–52.5]	46.0 [40.0–72.0]	0.15
mPAP (mmHg)	24.5 [18.5–33.5]	35.3 [26.7–44.0]	0.15
dPAP (mmHg)	19.0 [11.8–24.0]	28.0 [20.0–30.0]	0.28
PWP (mmHg)	18.0 [11.8–24.0]	28.0 [16.0–30.0]	0.28
TPG	7.7 [5.3–10.8]	11.0 [6.3–13.7]	0.11
CO (L/min)	4.1 [3.5–4.6]	3.9 [3.4–4.5]	0.54
PVR (WU)	1.9 [1.4–2.6]	3.1 [1.8–3.4]	0.45
Pulmonary arterial compliance (mL/mmHg)	2.9 [1.7–3.5]	1.7 [1.2–3.8]	0.32
Isolated RV failure after HT	8 (21.1%)	1 (9.1%)	0.37
Severe primary graft failure	7 (18.4%)	2 (18.2%)	0.96
Acute kidney injury after HT requiring RRT	6 (15.8%)	3 (27.3%)	0.41
Ischemic time	222.5 [153.8–260.0]	222.0 [120.0–241.0]	0.32
Length of stay in ICU (days)	8.0 [6.0–18.0]	6.0 [5.0–12.0]	0.92
HF admission after HT	3 (7.9%)	2 (18.2%)	0.30
Death after HT	8 (21.1%)	1 (9.1%)	0.38

Values are shown as mean ± standard deviation for parametric continuous variables and median (interquartile range) for nonparametric ones. CO, cardiac output; HF, heart failure; HT, heart transplant; ICU, intensive care unit; INTERMACS, Interagency Registry for Mechanically Assisted Circulatory Support; FAC, fractional area change; LVEF, left ventricle ejection fraction; NYHA, New York Heart Association; OCT, optical coherence tomography; PAAT, pulmonary artery acceleration time; s/m/dPAP, systolic/medium/diastolic pulmonary artery pressure; PWP, pulmonary wedge pressure; PVR, pulmonary vascular resistance; RA, right atrium; RRT, renal replacement therapy; RV, right ventricle; TAPSE, tricuspid annular plane systolic excursion; TPG, transpulmonary gradient.
